# Rates of colorectal cancer diagnosis and mortality in people with severe mental illness: results from Australia’s National Bowel Cancer Screening Programme

**DOI:** 10.1017/S2045796024000787

**Published:** 2024-12-17

**Authors:** S. Kisely, K. Spilsbury, C. Bull, S. Jordan, B.J. Kendall, D. Siskind, G. Sara, M. Protani, D. Lawrence

**Affiliations:** 1 Princess Alexandra Hospital Southside Clinical Unit, Greater Brisbane Clinical School, Medical School, The University of Queensland, Brisbane, QLD, Australia; 2 Metro South Addiction and Mental Health Service, Brisbane, QLD, Australia; 3 Departments of Psychiatry, Community Health and Epidemiology, Dalhousie University, Halifax, Nova Scotia, Canada; 4 The ALIVE National Centre for Mental Health Research Translation, The University of Queensland, Brisbane, QLD, Australia; 5 School of Public Health, Curtin University, Perth, Western Australia, Australia; 6 Institute for Health Research, University of Notre Dame Australia, Fremantle, Western Australia, Australia; 7 Queensland Centre for Mental Health Research, The University of Queensland, Brisbane, QLD, Australia; 8 School of Public Health, University of Queensland, Brisbane, QLD, Australia; 9 Population Health Department, QIMR Berghofer Medical Research Institute, Brisbane, QLD, Australia; 10 Department of Gastroenterology and Hepatology, Princess Alexandra Hospital, Brisbane, QLD, Australia; 11 InforMH, System Information and Analytics Branch, NSW Ministry of Health, Sydney, QLD, Australia; 12 Northern Clinical School, Faculty of Medicine and Health, University of Sydney, Sydney, NSW, Australia; 13 Faculty of Medicine and Health, University of New South Wales, Sydney, NSW, Australia

**Keywords:** cancer treatment, colorectal cancer, severe mental illness, treatment disparities, mortality, NBCSP, screening

## Abstract

**Aims:**

Studies show that people with severe mental illness (SMI) have a greater risk of dying from colorectal cancer (CRC). These studies mostly predate the introduction of national bowel cancer screening programmes (NBCSPs) and it is unknown if these have reduced disparity in CRC-related mortality for people with SMI.

**Methods:**

We compared mortality rates following CRC diagnosis at colonoscopy between a nationally representative sample of people with and without SMI who participated in Australia’s NBCSP. Participation was defined as the return of a valid immunochemical faecal occult blood test (iFOBT). We also compared mortality rates between people with SMI who did and did not participate in the NBCSP. SMI was defined as receiving two or more Pharmaceutical Benefits Scheme prescriptions for second-generation antipsychotics or lithium.

**Results:**

Amongst NBCSP participants, the incidence of CRC in the SMI cohort was lower than in the controls (hazard ratio [HR] 0.77, 95% confidence interval [CI] 0.61–0.98). In spite of this, their all-cause mortality rate was 1.84 times higher (95% CI 1.12–3.03), although there was only weak evidence of a difference in CRC-specific mortality (HR 1.82; 95% CI 0.93–3.57). People with SMI who participated in the NBCSP had better all-cause survival than those who were invited to participate but did not return a valid iFOBT (HR 0.67, 95% CI 0.50–0.88). The benefit of participation was strongest for males with SMI and included improved all-cause and CRC-specific survival.

**Conclusions:**

Participation in the NBCSP may be associated with improved survival following a CRC diagnosis for people with SMI, especially males, although they still experienced greater mortality than the general population. Approaches to improving CRC outcomes in people with SMI should include targeted screening, and increased awareness about the benefits or participation.

**Trial registration:**

Australian and New Zealand Clinical Trials Registry (Trial ID: ACTRN12620000781943).

## Introduction

Cancer is a leading cause of mortality in people with a range of psychiatric disorders, particularly severe mental illnesses (SMI) such as schizophrenia, bipolar and major depressive disorder (Kisely *et al.*, 2013a, 2008). Colorectal cancer (CRC) is one of the most common causes of cancer death amongst those with SMI (Crump *et al.*, 2013; Cunningham *et al.*, 2015; Kisely *et al.*, 2005; Lawrence *et al.*, 2000; Olfson *et al.*, 2015; Toender *et al.*, 2018), resulting in mortality rates that are up to 78% higher than the general population (Kisely *et al.*, 2013a, 2008; Lawrence *et al.*, 2013, 2000; Mahar *et al.*, 2020).

Many of these results predate the introduction of national bowel cancer screening programmes (NBCSPs) in countries such as England and Australia and it is unknown if these programmes have reduced the disparity in CRC-related mortality amongst people with SMI (Australian Institute of Health and Welfare, 2023; Koo *et al.*, 2017). Preliminary evidence suggests that the participation in screening programmes of people with SMI has been notably lower than that of the general population (Kirkøen *et al.*, 2023; Kisely *et al.*, 2024). For instance, in an Australian cohort study, people with SMI were less likely to return a valid immunochemical faecal occult blood test (iFOBT) but more likely to return a positive test result and less likely to undergo a follow-up colonoscopy (Kisely *et al.*, 2024). Lower rates of participation amongst individuals with SMI in the French national CRC screening programme have also been reported, as well as lower rates of follow-up colonoscopy (Seppänen *et al.*, 2024). However, the effects on subsequent care pathways in terms of mortality are not known.

We investigated care pathways following colonoscopy in the same Australian cohort as our previous work (Kisely *et al.*, 2024), as part of the Colorectal Cancer Outcomes in people with Severe Mental Illness Cohort (COSMIC) Study (Protani *et al.*, 2021). Specifically, this study had two aims: (i) to compare mortality rates following CRC diagnosis between NBCSP participants with positive iFOBT, with and without SMI; and (ii) to compare mortality rates following CRC diagnosis between people with SMI who did and did not initially participate in the NBCSP as defined by the return of a valid iFOBT.

## Methods

A staged roll-out of the Australian population-based NBCSP began in 2006. The programme is managed by the Commonwealth Government and targets Australians aged 50–74, who are sent an iFOBT screening kit every 2 years. Participants complete the test at home and mail it to the NBCSP pathology laboratory for analysis. Results are shared with the participant, their general practitioner (GP) and the National Cancer Screening Register. Those with positive results are advised to consult their GP for further assessment, typically a colonoscopy.

COSMIC is an observational cohort study of the NBCSP that uses de-identified linked administrative data. Ethics approval was obtained from The University of Queensland Human Research Ethics Committee (2019000296) and the Australian Institute of Health and Welfare Ethics Committee (E2019-5-1108). We published a pre-study protocol and prospectively registered the project with the Australian and New Zealand Clinical Trials Registry (Trial No. ACTRN12620000781943) (Protani *et al.*, 2021). The STROBE guidelines were followed (von Elm *et al.*, 2008).

### Defining the eligible cohorts

Pharmaceutical Benefits Scheme (PBS) data were used to identify eligible COSMIC cohorts. In Australia, atypical antipsychotic medications and lithium are the primary treatments for SMI. PBS prescriptions for atypical antipsychotics necessitate a specific authority code indicating whether the condition being treated is schizophrenia or bipolar affective disorder while lithium is predominantly used for the latter although it also sometimes prescribed for treatment-resistant depression (Malhi *et al.*, 2021). The use of lithium in other conditions such as neurodegenerative conditions and cluster headache is off-label or experimental (Lund *et al.*, 2023; Singh *et al.*, 2023).

All Australians aged 50–74 years at 01/01/2006 or who turned 50 after this date with any history of being dispensed lithium or, atypical antipsychotic medications were eligible for inclusion in the SMI cohort. A person was classified as having SMI if they were dispensed two or more prescriptions at therapeutic doses for these conditions within a 12-month period. In relation to the controls, the data custodians would not allow access to the full population dataset as the control group. We therefore randomly selected nine people for every one classified as having SMI of the same age range who had never been prescribed psychotropic medications (anti-psychotic, lithium or anti-depressant medications). These individuals were identified in the Medicare Enrolments File, which contains all Australian residents eligible for Medicare benefits. Medicare is Australia’s universal healthcare scheme that provides access to government subsidised medical services (via the Medicare Benefits Scheme – MBS) and prescriptions (via the PBS) for all citizens and permanent residents.

Inclusion criteria for both the SMI and control cohorts were an invitation to participate in the NBCSP from 01/08/2006 to 31/12/2016. Phased implementation of NBCSP began in 2006. Currently, all Australians aged between 50 and 74 years who have a postal address registered with Medicare (Australia’s universal healthcare system) are invited to participate in the NBCSP and are mailed a free home-based iFOBT test every 2 years. We defined participation as the return of a valid iFOBT. By following these individuals over time, we identified and included any subsequent NBCSP invitations occurring prior to 31/12/2016; positive iFOBTs, colonoscopies and CRC diagnoses occurring prior to 31/12/2017; and deaths occurring prior to 31/07/2021. This enabled comparative analysis and adequate follow-up before the COVID-19 pandemic.

We excluded participants from the control cohort if they had a history of antidepressant use. We also excluded any participants with a history of CRC that preceded the date of first NBCSP invitation. We did not exclude participants if they had a prior history of other cancer types. People who met SMI cohort eligibility (two prescriptions within a 12-month period) after the date of their first NBCSP invitation were also excluded from the SMI cohort.

Information available in the NBCSP dataset included socio-demographic variables, round of invitation, details of participation (those who returned valid and non-valid iFOBT tests) and whether a follow-up colonoscopy after positive iFOBT was recorded. Each participants’ subsequent care pathways were mapped using the NBCSP database, supplemented with information from the MBS, National Death Index (NDI) and Australian Cancer Database (ACD).

### Outcome measures

Outcomes of interest for this study were all-cause and cause-specific mortality rates following diagnosis CRC at colonoscopy. Colonoscopies were not always reported to the NBCSP and so we also included colonoscopies recorded in the MBS data within the relevant date ranges (service item numbers 32084, 32087–32090, 32093, 32084, 32087, 32222–32229). Colonoscopies were only included if they followed a positive iFOBT from the NBCSP and occurred before any subsequent NBCSP invitation or within 2 years of the latest invitation or before 31/12/2016, to allow a 1-year follow up period to identify subsequent CRC.

Cancer is a notifiable disease in Australia, so the complete cancer history for both cohorts was identifiable from 1982 to 2017 through the ACD. International Classification of Diseases for Oncology (ICD-O) topography codes C18, C19, C20 and C21 with the exclusion of morphology codes 959–998 (indicating lymphomas) were used to identify CRC. We included C21 anal tumours, because these were detected by the NBCSP.

Date and cause of death coded (ICD10) data were obtained from the NDI with records available up to 01/07/2021. A CRC-specific death was defined as CRC (C18, C19, C20 and C21) being the underlying or a contributory cause of death, as noted in the death certificate. An expanded definition of CRC-specific deaths that included secondary malignant neoplasms to the brain, liver, lung, peritoneum and abdominal/thoracic/pelvic lymph nodes was also assessed. Due to administrative delays in coding cause of death from death certificates, cause of death information was not available in 39% and 54% of deaths that occurred in 2020 and 2021, respectively. We classified all uncoded causes of death as being from ‘other’ causes. Subsequently, we also conducted a sensitivity analysis to assess for any bias of being unable to identify CRC as cause of death in later deaths by censoring the survival analysis 31/12/2019.

### Covariates

We included the following covariates in our models: sex, age at NBCSP invitation, geographic access to services, socio-economic status, state of residence and calendar year of invitation or diagnosis. Geographic accessibility to services was classified according to whether participants lived in major urban cities, inner regional, outer regional and combined remote or very remote areas of Australia using their area of residence at time of NBCSP invitation. Socio-economic status was assessed using quintiles of the index of relative socio-economic disadvantage based on area of residence (SEIFA) (Australian Bureau of Statistics, 2023). This is because participation in the NBCSP declines with increasing social disadvantage and geographical remoteness (Australian Institute of Health and Welfare, 2023). Indigenous status was included in models but not separately reported because of restrictions on data release. Additional CRC-related covariates included whether polypectomy was performed at colonoscopy, tumour site (categorised as proximal colon, splenic flexure, distal colon, rectosigmoid junction, rectum and other [multiple/overlapping sites and anal]) and broad morphology characteristics (mucinous adenocarcinoma, adenocarcinoma in adenoma and other). Cancer staging information was not available. Finally, we investigated any differences in mortality between people with SMI who returned a valid iFOBT sample and those who were invited to participate but did not return a valid iFOBT.

### Statistical analysis

Demographic data for the general population relative to individuals with SMI were described using means, standard deviations, standardised mean differences (SMDs) and proportions. Unadjusted relative risks (RRs) were estimated using Poisson regression and mean differences with *t*-tests.

For time-to-event analysis, Cox proportional hazards regression models were used to estimate the unadjusted and adjusted relative hazard rates of CRC diagnosis and all-cause and CRC-specific mortality. For time-to-CRC diagnosis, only cancers diagnosed within 1 year of colonoscopy were modelled. For time-to all-cause-mortality, a person was considered at risk of death from the date of their CRC diagnosis until death or the censor date (01/07/2021). Cause-specific mortality was assessed under competing risks frameworks. For cause-specific hazards, censoring on the competing risk was used because only the relative rates by SMI status were of interest.

For time-to-event models, Schoenfeld residuals, -ln[-ln(survival)] plots and tests based on time interactions were used to assess proportionality of the hazard and residuals. Link tests were used to assess goodness of fit. Plausible interaction terms were assessed by Wald tests. All analysis was performed using Stata 18 (StataCorp, 2023).

## Results

### Cohort overview

**Figure 1.**
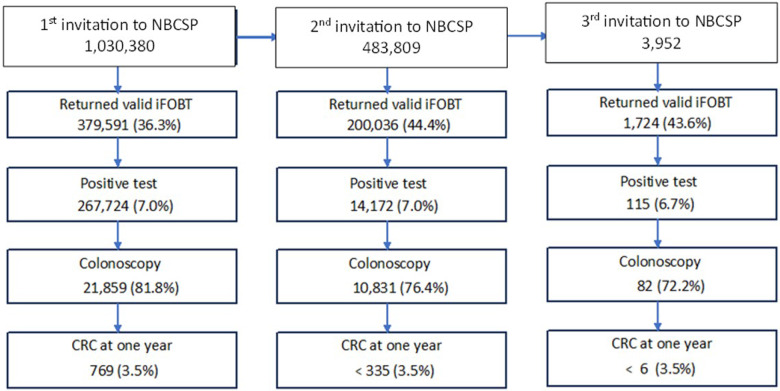
Schematic representation of participant pathways through the NBCSP from 2006-2016 relative to invitation round.

Over 1 million people in the present cohort received an invitation to participate in the NBCSP between 2006 and 2016 (Fig. 1). As we used a 1:9 sampling ratio, approximately 10% of the study cohort (*n* = 98,028) were individuals with SMI and 932,352 the selected controls (90.5%). Of the total cohort, 374,591 (36.4%) returned a valid iFOBT following the first NBCSP invitation, and 26,724 (7.1%) had a positive iFOBT indicating blood in the stool. Of those with a positive iFOBT, 21,859 (81.8%) had a colonoscopy and 769 (3.5%) were diagnosed with CRC within 1 year.

Table 1 shows that participants with SMI who underwent a colonoscopy following a positive iFOBT were more likely to be younger, female and experience greater levels of socio-economic disadvantage or live in more remote areas compared to participants without SMI. Individuals with SMI also had an increased number of days between reporting of their positive iFOBT results and having a colonoscopy, and were less likely to participate in the NBCSP after their first invitation. They were less likely to have polyps removed at colonoscopy compared to individuals without SMI (33.0% vs 36.2%; RR 0.91, 95% confidence interval [CI] 0.86–0.96).

**Table 1.** Characteristics of NBCSP participants who underwent colonoscopy following a positive iFOBT^a^, by SMI status

	**No SMI** *n* = 29,338		**SMI** *n* = 3,021		
	**Mean**	**SD**	**Mean**	**SD**	**SMD [95% CI]**
Age at NBCSP invite (years)	59.6	7.0	58.2	6.8	−1.4 [−1.6–1.1]
Days from +ve iFOBT to colonoscopy	89.8	142.7	111.7	153.1	21.9 [16.5–27.3]
Days from NBCSP invite to colonoscopy	170.4	212.7	186.3	189.8	16.4 [8.6–24.4]
	***n***	**%**	***n***	**%**	**Unadjusted** **RR [95% CI]**^c^
**Year of NBCSP invitation**					
2006−2009	5,479	18.7	423	14.0	Ref
2010−2013	10,570	36.0	1,235	40.9	1.46 [1.31–1.63]
2014−2016	13,289	45.3	1,363	45.1	1.30 [1.16–1.45]
**NBSCP invitation round**^b^					
1	19,296	65.8	2,147	71.1	Ref
2+	10,042	34.3	874	28.9	0.80 [0.74–0.87]
**Sex**					
Male	17,051	58.1	1,403	46.5	Ref
Female	12,287	41.9	1,618	53.5	1.53 [1.42–1.64]
**Index of relative disadvantage**^d^					
Least disadvantage	7,815	26.6	561	18.6	Ref
Less disadvantage	6,183	21.1	576	19.1	1.27 [1.13–1.43]
Median disadvantage	5,707	19.5	587	19.4	1.39 [1.24–1.56]
More disadvantaged	5,012	17.1	577	19.1	1.54 [1.37–1.73]
Most disadvantaged	4,280	14.6	678	22.4	2.04 [1.82–2.28]
**Remoteness classification**					
Major cities	20,448	69.7	2,087	69.1	Ref
Inner regional	5,571	19.0	615	20.4	1.07 [0.98–1.18]
Outer regional	2,620	8.9	247	8.2	0.93 [0.81–1.06]
Remote	245	0.8	18	0.6	0.74 [0.46–1.18]
Very remote	109	0.4	9	0.3	0.82 [0.43–1.59]
Unknown	345	1.2	45	1.5	1.25 [0.93–2.67]

a There were 415 participants who had colonoscopies at multiple NBSCP rounds; only characteristics of their most recent colonoscopy were included in this table.

b Due to privacy concerns from small numbers, the number of subsequent NBCSP invitations were collapsed into a single category.

c Estimated from univariate Poisson regression models.

d Unknown in 341.

SMI, severe mental illness; SMD, standardised mean difference; CI, confidence interval; RR, relative risk; SD, standard deviation; iFOBT, immunochemical faecal occult blood test; CRC, colorectal cancer.

### CRC diagnosis rates within 1 year of colonoscopy following a positive iFOBT

Of the 32,359 NBCSP participants who had a colonoscopy following positive iFOBT after any round of invitation, 1,105 (3.4%) were diagnosed with CRC within 1 year (Fig. 1). Of these, 75 (2.4%) participants had SMI and 1,030 (3.4%) did not (hazard ratio [HR] 0.70, 95%CI 0.55–0.88). After adjusting for age, sex, year of NBCSP invitation, history of previous non-CRC cancer, index of relative disadvantage, residential remoteness and NBCSP round, CRC incidence in the SMI cohort remained lower (HR 0.77, 95% CI 0.61–0.98).

### Mortality following CRC diagnosis at colonoscopy in NBCSP participants

Table 2 shows that of the 1,105 NBCSP participants diagnosed with CRC within 1 year of colonoscopy following positive iFOBT, 18 (24.0%) SMI participants died from any cause compared to 145 (14.1%) without SMI. This equated to an adjusted all-cause mortality HR of 1.84 (95%CI 1.12–3.03). Where the cause of death was specifically listed as CRC (*n* = 10 SMI participants and *n* = 85 without SMI), the adjusted HR was 1.66 (95% CI 0.85–3.25). Sensitivity analysis restricting the follow-up date to 31/12/2019 indicated a stronger association of SMI with CRC-specific mortality (HR 1.82, 95% CI 0.93–3.57) when the potential bias of the high proportion of uncoded causes of death in 2020/21 were excluded. Expanding the definition of CRC-specific deaths to include deaths from secondary malignant neoplasms of common CRC metastatic sites did not greatly alter the results (Table 2).

**Table 2.** Unadjusted and adjusted all-cause and CRC-specific mortality rates for the NBCSP participants diagnosed with CRC within 1 year of colonoscopy following positive iFOBT after any round of invitation to the NBCSP, by SMI status

	**No SMI** *n* = 1,030		**SMI** *n* = 75							
**Mortality**	***n***	**%**	***n***	**%**	**HR**	**95% CI**	***P*-value**	**HR_Adj_**^a^	**95% CI**	***P*-value**
**All-causes**	145	14.1	18	24.0	1.82	1.11−2.96	0.017	1.84	1.12−3.03	0.017
**CRC-specific**										
CRC-specific^b^	85	8.3	10	13.3	1.70	0.88−3.28	0.111	1.66	0.85−3.25	0.135
CRC-specific^b^^,^^c^	77	7.5	10	13.3	1.87	0.97−3.61	0.063	1.82	0.93−3.57	0.081
**CRC-specific** – **includes secondary neoplasms**										
CRC-specific^b^^,^^d^	92	8.9	10	13.3	1.58	0.82−3.03	0.172	1.50	0.77−2.91	0.235
CRC-specific^b^^–^^d^	83	8.1	10	13.3	1.74	0.90−3.35	0.099	1.64	0.84−3.20	0.146

a Adjusted for age at diagnosis, sex, calendar year, Indigenous status, relative social disadvantage, polypectomy, tumour morphology and site.

b Cause specific proportional hazards models censoring on the alternative cause of death.

c Sensitivity analysis restricting survival follow-up to 31 December 2019 to remove deaths of uncoded cause due to administrative lag.

d Sensitivity analysis that includes secondary malignant neoplasms of liver, lungs, brain, lymph nodes and peritoneum.

HR, hazard ratio; SMI, severe mental illness; CRC, colorectal cancer.

### Mortality following CRC diagnosis in individuals with SMI who did and did not participate in the NBCSP

Of the 98,028 people with SMI invited to participate in the NBCSP, 669 (0.7%) individuals were subsequently diagnosed with CRC before 31/12/2017. Only 25% (*n* = 170) of these individuals had participated in the NBCSP by returning a valid iFOBT sample following their most recent NBCSP invitation prior to CRC diagnosis. Amongst the 669 people with SMI diagnosed with CRC, significantly better all-cause survival (HR 0.67, 95% CI 0.50–0.88) was observed for those who participated in the NBCSP compared to those who did not (Table 3). However, this survival advantage varied significantly by sex. Males with SMI who participated in the NBCSP prior to CRC diagnosis had almost half the all-cause and CRC-specific mortality rate of males with SMI who did not participate in the NBCSP prior to diagnosis. There was no difference in all cause or CRC-specific mortality outcomes between females with SMI who participated and females who did not participate in the NBCSP.

**Table 3.** Adjusted relative hazard of all-cause mortality and CRC-specific mortality in people with SMI diagnosed with CRC who participated in the NBCSP compared to those who did not participate by sex

	**All-cause mortality**			**CRC-specific mortality**^a^		
	**HR**	**95% CI**	***P*-value**	**HR**	**95% CI**	***P*-value**
**Overall**						
Participant vs non-participant	0.67	0.50–0.88	0.005	0.74	0.52–1.06	0.097
Participation × sex interaction						
**Males**						
Participant vs non-participant	0.51	0.34–0.75	0.001	0.54	0.33–0.89	0.015
**Females**						
Participant vs non-participant	0.97	0.64–1.47	0.888	1.14	0.67–1.93	0.628

All models adjusted for age at diagnosis, sex, calendar year, relative social disadvantage, accessibility to services, tumour morphology and site with sex included as an interaction term with NBCSP participation status.

a Modelled on the log cumulative hazard scale with death from other causes censored.

HR, hazard ratio; CRC, colorectal cancer.

## Discussion

To our knowledge, this is the first Australia-wide study to report on mortality following a CRC diagnosis after the introduction of the NBCSP. The rate of CRC diagnosis in people with SMI following colonoscopy as part of the NBCSP was lower than that of the non-SMI cohort. However, subsequent all-cause mortality was significantly higher, and CRC-specific mortality rates were non-significantly greater. This finding, where CRC incidence amongst individuals with SMI is the same or lower than that of people without SMI despite a higher mortality rate, is also observed in population-based studies beyond NBCSP participants (Kisely *et al.*, 2013a, 2008; Lawrence *et al.*, 2000). In the case of schizophrenia, a meta-analysis of 16 large cohort studies found that CRC incidence was actually lower than that of the general population, especially in males (Li *et al.*, 2018).

One explanation for a higher mortality and lower incidence rate is that people with SMI have lower screening participation rates. This would be consistent with the finding from the present study that people with SMI who returned a valid iFOBT sample had better all-cause survival than those who were invited to participate but did not return a valid iFOBT. In particular, the benefit of participation was strongest for males with SMI and included improved all-cause and CRC-specific survival.

Another explanation for increased mortality following lower incidence is that people with SMI present with more advanced cancer. Although information on cancer staging was not available in this sample, people with SMI were less likely to have polyps removed at colonoscopy, which may be an indicator that they were presenting with more advanced cancer. In addition, previous work has shown that the presence of metastases on presentation is statistically more likely in people with a history of psychiatric disorder (Kisely *et al.*, 2013a).

There may be other reasons. A recent systematic review suggested that people with SMI had a reduced likelihood of curative treatment such as surgery or adjuvant chemo- or radiotherapy, although this information comes from clinical rather than population-based samples (Protani *et al.*, 2022). There may also be barriers to health service access as a result of limited financial resources, difficulties in obtaining private health cover or negative attitudes on the part of treating clinicians (Ostrow *et al.*, 2014; Thornicroft, 2008). The findings from the present study suggest that the same factors may apply to NBCSP participants with SMI. Factors other than cancer may also contribute to the increased case fatality seen in people with SMI. For instance, SMI is associated with greater levels of physical comorbidity such as cardiovascular disease, chronic obstructive pulmonary disease, obesity and diabetes, all of which could contribute to all-cause mortality (Janssen *et al.*, 2015; Viron and Stern, 2010). This is compounded by that fact that people with SMI face barriers to equitable access to care for these conditions. Another possibility is that antipsychotic-induced constipation may have increased the risk of a positive iFOBT in the absence of CRC resulting in fewer diagnoses at colonoscopy even though people with SMI were less likely to undergo the procedure (Xu *et al.*, 2021). Finally, a Danish cohort study reported that compared to people without mental disorders, people with SMI are twice as likely to have an incomplete screening colonoscopy due to factors including poor bowel preparation, pain and technical difficulties (Thomsen *et al.*, 2023).

This study has several limitations arising from the use of linked administrative data. We relied on prescriptions instead of medical records to identify SMI, and so our case definition was limited to people prescribed lithium or atypical antipsychotic agents. Although there is no universal agreement on the meaning of SMI, some definitions include other conditions such as major depressive disorder (Gonzales *et al.*, 2022). However, broadening the scope to include these diagnoses would be impossible using the PBS given the widespread use of other psychotropic agents such as antidepressants for milder mental illness. Our case definition may also have overlooked individuals with SMI who received private prescriptions or were not prescribed any medications. Nonetheless, such cases represent a small fraction of those with SMI. For instance, an Australian survey of adults with psychotic illness under community mental health services revealed that 81.6% were prescribed antipsychotic medications (Morgan *et al.*, 2012). Moreover, private prescriptions account for less than 10% of all community prescription medications.

Authority codes may also have been inappropriately applied and do not encompass first-generation antipsychotics (FGAs). However, FGAs make up less than 14% of dispensed antipsychotics according to recent data (Taylor *et al.*, 2021). Overall, the effect of any misclassification would be to underestimate the disparity between people with and without SMI, thus rendering our estimates more conservative. We deliberately excluded individuals from the control group if they were prescribed antidepressants as our specific aim was to compare the outcomes of NBSCP participants with psychosis or bipolar affective disorder to that in the general population. However, this may limit the real-world generalisability of our findings given that roughly 15% of Australians are prescribed antidepressants annually (Australian Institute of Health and Welfare, 2024).

We were unable to check for the presence of SMI as identified through the PBS with Medicare item numbers for contacts with psychiatrists, GPs and other clinicians as these do not contain diagnoses. Similarly, admissions to hospitals are recorded in the administrative databases held at state or territorial level and require data custodian approval in each of Australia’s eight jurisdictions. This also meant that we were unable to explore care pathways from the incidence of CRC through to mortality. Another limitation is that the stage of CRC at diagnosis is not routinely collected by all state or territory cancer registries, and so this information is not available in the ACD. We therefore plan to undertake a study limited to New South Wales (NSW) where staging is recorded, linking this to the NSW Admitted Patient Data Collection (APDC) for information on subsequent cancer care (Protani *et al.*, 2021).

There was also no information on important covariates such as ethnicity, country of birth, marital status, education level, comorbidity and social functioning. Moreover, socio-economic status was determined based on postcode rather than individual-level data. We were also unable to adjust for the impact of lifestyle factors such as diet, or tobacco, alcohol and substance use. Nonetheless, it is possible that CRC diagnosis rates might have been even lower if lifestyle factors had been considered, potentially reinforcing our conclusion that individuals with SMI are not inherently more susceptible to cancer but are at a heightened risk of mortality from it. We were also not able to adjust for comorbidity such as cardiovascular disease, chronic obstructive pulmonary disease, obesity and diabetes). As noted previously, these conditions are more likely to be present in people with SMI, influencing both treatment and subsequent prognosis (Gross *et al.*, 2007; Janssen *et al.*, 2015; Viron and Stern, 2010).

Furthermore, while all-cause mortality was significantly greater than the general population, our study may have been underpowered to detect differences in CRC-specific rates. Finally, the improved survival seen in males with SMI who were NBCSP participants compared to non-participants may be due to explanations other than the programme. Non-participants may have had different lifestyle factors, fewer other preventative interventions and less overall healthcare contact.

Notwithstanding these limitations, our results may indicate the need for greater action to improve cancer outcomes for people with SMI. These strategies should involve multi-faceted approaches at both the individual and health systems level. Firstly, increased awareness by healthcare providers about the increased risk of mortality from CRC is crucial. This includes promoting regular screening and early detection initiatives tailored to this population. Additionally, establishing collaborative care models that integrate mental health services with oncology and primary care can ensure comprehensive and coordinated management throughout the cancer care continuum (Irwin *et al.*, 2014). This might involve deploying navigators or care coordinators to support individuals with psychiatric disorders through diagnosis, treatment decision-making and follow-up care (Irwin *et al.*, 2014). Improved community or outpatient follow-up by mental health teams can lead to reductions in all-cause mortality (Kisely *et al.*, 2013b), as well as the reengagement of people with SMI who have been lost to psychiatric follow-up (Bowersox *et al.*, 2012; Irwin *et al.*, 2014). Addressing systemic barriers such as stigma, patient mistrust, clinician training deficiencies and healthcare fragmentation between psychiatric and oncological services is also important. This might include the destigmatisation of mental illness, improved patient–provider communication and appropriate training for healthcare professionals in managing comorbidities (Grassi and Riba, 2021). Finally, further research is indicated to identify specific challenges and interventions along the CRC care pathway for individuals with psychiatric disorders (Protani *et al.*, 2021). This includes exploring the impact of socio-economic factors, access to care and individual experiences on treatment adherence and outcomes through both quantitative and qualitative approaches.

## Supporting information

Kisely et al. supplementary material Kisely et al. supplementary material

## Data Availability

The data for this study will not be shared, as we do not have permission from the data custodians or ethics approval to do so.

## Supplementary material

The supplementary material for this article can be found at https://doi.org/10.1017/S2045796024000787.
